# Advances in Research on Food Bioactive Molecules and Health

**DOI:** 10.3390/molecules26247678

**Published:** 2021-12-19

**Authors:** Mohamed Fawzy Ramadan, Alessandra Durazzo, Massimo Lucarini

**Affiliations:** 1 Deanship of Scientific Research, Umm Al-Qura University, Makkah 24381, Saudi Arabia; 2 Agricultural Biochemistry Department, Faculty of Agriculture, Zagazig University, Zagazig 44519, Egypt; 3 CREA-Research Centre for Food and Nutrition, Via Ardeatina 546, 00178 Rome, Italy

**Keywords:** functional food, antioxidants, health-promoting effects, database, phytotherapy, phenolic compounds, extraction, analysis, phytochemicals, structure-function-relationship, health, disease

## Abstract

Fresh and processed food products are rich in bioactive molecules, including polysaccharides, vitamins, carotenoids, peptides, antioxidants, phenolics, phytosterols, and novel lipids. Bioactive molecules in food could prevent several diseases (i.e., metabolic syndrome, cardiovascular diseases, cancer, etc.). Thus, consumer awareness is growing about the health-promoting impact of food bioactive molecules. Health claims are essential added-value features, wherein health-enhancing potential of bioactives depend on their chemical structure. On the other hand, the investigation of the structure-function relationship of food bioactive molecules is of importance. In this regard, Molecules is delighted to highlight the importance of food bioactive molecules and their effect on health. In this Special Issue of Molecules, researchers are invited to contribute original research and up-to-date reviews.

Fresh and processed food products (vegetables, fruits, cereal, and dairy products) are rich in bioactive molecules, including vitamins, carotenoids, polysaccharides, proteins, peptides, antioxidants, phenolic compounds, sterols, and bioactive lipids [1,2,3,4,5,6,7]. Some fermented food products are also considered novel items with health benefits [8,9,10]. In addition, many bioactive molecules in food have a synergistic impact with medicaments [11,12,13] and prevent several diseases (i.e., metabolic syndrome, cardiovascular diseases, cancer, etc.).

Consumer awareness is growing about the health-enhancing effects of food bioactive molecules from plant and animals sources. Health claims are important value-added features for consumers, wherein authorities accept health claims in functional foods based on scientific evidence. The health-promoting impacts of bioactive compounds depend on their chemical structure; therefore, novel analytical techniques have been developed to elucidate the structure of active molecules [14,15,16,17]. Besides, novel techniques have been developed to increase the yield of bioactive molecules. On the other hand, investigating the structure-function relationship of food bioactive molecules is essential. Several factors might affect the structure-function relationship of food bioactives, including agricultural practices, cultivars, post-harvest treatments, processing, and storage conditions [18,19,20,21,22,23,24].

To give a recent view of the interest raised in the international research context within this topic, a search throughout the Scopus database was carried out using a string TITLE-ABS-KEY (“food *”, “health *”, and “disease *”). The search returned 87,471 documents covering the time range from 1884 to 2022. In the last ten years (from 2011–2020), the search returned 48,842 documents (Figure 1).

Apart from the published documents, approx. 30,600 were research articles, 1160 conference papers, 10,500 reviews, and 2800 book chapters. The documents annually published on “food *”, “health *”, and “disease *” are notably increased from 3537 contributions in 2011 to 7370 contributions in 2020. This measurable indicator reflects the importance and interest in “food *”, “health *”, and “disease *” as a topic in the scientific community. The documents are related to the subject areas of Medicine, Agricultural and Biological Sciences, Biochemistry, Genetics, and Molecular Biology, Nursing, Immunology and Microbiology, Pharmacology, Toxicology, and Pharmaceutics, and Environmental Science. Scientists from the USA, UK, China, India, and Australia emerged as main authors.

This Special Issue of Molecules is entitled Advances in Research on Food Bioactive Molecules and Health. In this regard, Molecules is delighted to highlight the importance of food bioactive molecules and their effect on health. Researchers from different fields, including food chemistry, biochemistry, natural products, phytotherapy, pharmacology, medicine, and biotechnology, are expected to disseminate their results in this issue. In this Special Issue of Molecules, researchers are invited to contribute original, unpublished research and up-to-date review articles that analyze and describe bioactive molecules in fresh and processed food products; their stability during food processing and storage; and the mechanisms of their digestion, bioactivity (in vitro and in vivo), and metabolite formation. In addition, the impact of food bioactive molecules in preventing and treating diseases is of interest.

Potential topics include, but are not limited to, the following: (i) novel analytical techniques in the structure elucidation of food bioactive molecules; (ii) chemistry and functionality of food bioactive molecules; (iii) factors affecting the structure-function relationship of food bioactive molecules; (iv) the effect of industrial and biotechnological processing on food bioactive molecules; (v) modification of food bioactive molecules to enhance their health-promoting effects.

## Figures and Tables

**Figure 1:**
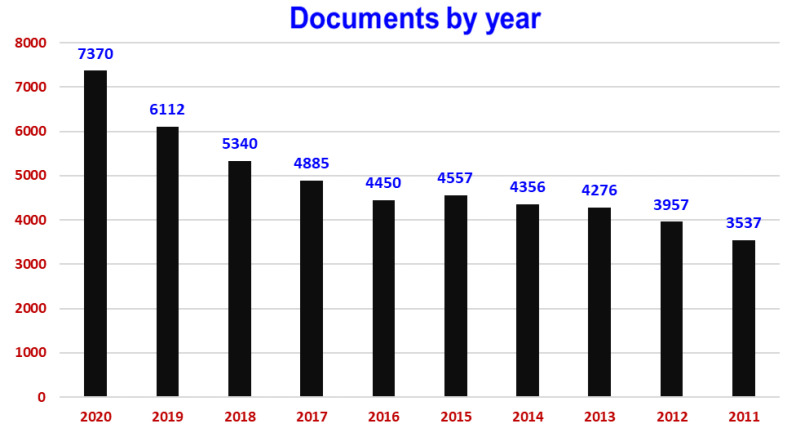
Scholarly output on “food *”, “health *”, and “disease *” from 2011–2020 (www.scopus.com).
